# Solidification Rate as Key Factor in Strengthening Mechanisms, Tensile Properties, and Phase Features in Cast Al-Mg-Sc Alloys

**DOI:** 10.3390/ma19040796

**Published:** 2026-02-18

**Authors:** Anderson Thadeu Nunes, José Eduardo Spinelli

**Affiliations:** Department of Materials Engineering, Federal University of São Carlos, São Carlos 13565-905, SP, Brazil; atnunes@estudante.ufscar.br

**Keywords:** Al-Mg-Sc, solidification, Al_3_Sc, mechanical properties, PLC

## Abstract

Scandium (Sc), when added together with magnesium (Mg), forms a highly effective synergistic pair in aluminum (Al) alloys, enhancing their performance in various applications. While the thermomechanical processing and heat treatment of such Al-Mg-Sc alloys have been well investigated, the behavior and features of their as-cast state remain less understood. In particular, the evolution of cellular/dendritic microstructures and the formation of phases at submicrometric and nanometric scales, especially those developing during solid-state cooling, require further elucidation. The present study employs a combination of conventional and advanced characterization techniques in the Al-5 wt.%Mg-0.4 wt.% Sc alloy, including CALPHAD, optical microscopy, scanning electron microscopy (SEM), transmission and scanning transmission electron microscopy (TEM/STEM) with energy-dispersive spectroscopy (EDS), x-ray diffractometry (XRD), tensile testing, and fractographic analysis. Al-rich dendrites surrounded by Al_3_Sc, AlFe, and β-Al_3_Mg_2_ phases and the formation of primary submicrometric clusters containing AlFe and Al_3_Sc have been identified, revealing important microstructural features that depend strongly on the solidification conditions. Moreover, nanometric Al_3_Sc precipitates mainly in the form of rod-like structures with sizes in the order of 50–200 nm have been observed within the α-Al matrix during solid-state cooling stage. At higher solidification rates, such as 15.3 °C/s, these precipitates remain predominantly in solid solution, indicating strong solidification rate dependence in the precipitation behavior. Comparisons between alloys containing 0.1 Sc and 0.4 Sc have demonstrated that the morphology, size, and distribution of Sc-rich phases significantly affect the stress–strain tensile response and underlying strengthening mechanisms. Distinct Portevin–Le Chatelier (PLC) effects have been observed, corresponding to very different serration activities in the stress–strain curves comparing both Al-5%Mg-0.4%Sc and Al-5%Mg-0.1%Sc alloy samples. Among the compositions and conditions studied, the Al–5Mg–0.4Sc alloy samples solidified under the fast-cooling condition (11.2 °C/s) exhibited the most improved mechanical performance, attaining a strength of 306 MPa and an elongation of 22.6%, underscoring the pivotal role of Sc content and solidification rate in achieving optimized mechanical properties.

## 1. Introduction

Aluminum (Al) and its alloys are widely used in engineering applications due to their combination of desirable properties, such as low density, high mechanical strength, excellent corrosion resistance, and high thermal and electrical conductivity [1]. Beyond the cited characteristics, the abundance of Al in the Earth’s crust in the form of bauxite has also contributed to its broad utilization, ranging from domestic utensils to high-demand applications in the aeronautical industry, microelectronics sector, and the manufacturing of aerospace components. Within this context, the development of new, stronger, and lighter Al alloys remains a pressing objective in materials engineering.

Ternary Al-Mg-Sc alloys present a promising pathway by combining the attractive properties of binary Al-Mg alloys with the beneficial effects of scandium (Sc). Sc can promote grain refinement, modify dendritic or cellular structures during solidification, and enable the formation of the Al_3_Sc phase in nanometric size during the post-solidification stage [2]. These effects result in alloys with enhanced mechanical strength, making them particularly suitable for structural applications where performance and weight are critical. In the search for lightweight alternatives for aircraft fuselage skin sheets, recent developments have emphasized Al-Mg-Sc alloys such as AA5024 and AA5028, containing 0.1–0.4 wt.% Sc [3]. These alloys are typically required to operate under combined mechanical loading and thermal cycling, often at service temperatures up to approximately 150–250 °C, where microstructural stability and strength retention are critical. For example, Scalmalloy^®^ (Al-Mg-Sc) chemistries have been employed in satellite parts, being advised to have a limit temperature of operation of about 130 °C [4]. These advancements highlight the potential of Sc additions to enhance the performance limits of Al-Mg-based alloys for demanding structural applications.

In the automotive, aeronautical, and aerospace sectors, specific strength, defined as the ratio between mechanical strength and density, is a key design parameter, since the use of strong yet lightweight materials directly influences fuel efficiency and overall energy performance. The Al-5%Mg-Sc alloys can meet these demands by offering low density alongside elevated mechanical strength, supported by the combined strengthening effects of Mg and Sc.

Processing routes for Al–Mg–Sc alloys encompass both conventional methods, applied individually or in combination, such as casting and heat treatment, as well as advanced laser-based techniques, including additive manufacturing and surface remelting. These methods allow the production of components with complex geometries and varied microstructures, largely due to the wide range of cooling rates involved. Given this, investigating new Al-Mg-Sc alloys under different processing conditions is essential to optimize mechanical properties and fulfill the stringent requirements of critical applications in aerospace and aviation sectors [5,6,7].

The Al_3_Sc precipitate can be coherent with the α-Al matrix and acts as a barrier to dislocation motion, significantly increasing the mechanical strength of the material. Moreover, Sc is a widely employed strategy to refine grains and promote the formation of strengthening precipitates. Both effects can be further enhanced through either controlled thermal treatments or controlled post-solidification cooling down. Sc may contribute to strengthening Al-Mg alloys both by refining the grain structure and by facilitating interactions between Al_3_Sc precipitates and dislocations within the Al-rich matrix.

In binary Al-Sc alloys, Al_3_Sc precipitates exhibit a coherent L1_2_ structure and typically nucleate as spherical nanoparticles during aging or remaining cooling down after solidification stage, providing significant strengthening via dislocation shearing. Under prolonged exposure or elevated temperatures, these precipitates may coarsen into cuboidal or faceted morphologies while retaining thermal stability due to the low diffusivity of Sc in Al [8,9,10]. The morphology of the Al_3_Sc is much less known for ternary and multicomponent Sc-containing alloys.

The study by Zhang et al. [11] reported the exceptional tensile strength in LPBF Al–Mn–Mg–Sc–Zr alloys, emphasizing thermally stable Al_3_Sc nano-precipitates and high-temperature performance. It explored microstructural anisotropy, peak-aging responses, and how nanoscale dispersoids influence elevated-temperature strength retention. Peak-aged samples contained stable nanoscale Al_3_Sc dispersoids uniformly distributed within a bimodal grain structure, contributing to fine-scale precipitation strengthening. Additionally, the mechanical testing revealed yield strength decreased with increasing test temperature, yet Al_3_Sc nano-precipitates remained stable and preserved some strengthening effect.

Al–Mg–Sc alloys are recognized for their superior combination of strength, corrosion resistance, and thermal stability, which have been attributed to the formation of coherent L1_2_-structured Al_3_Sc precipitates that hinder dislocation motion and grain coarsening [12]. These alloys have recently played a fundamental role in the advancement of rapid solidification and additive manufacturing techniques. In these cases, thermal history may strongly impact microstructure evolution and phase stability [13].

During solidification, local cooling rate strongly dictates dendritic arm spacing, solute distribution, and the initial nucleation of Al_3_Sc particles. Studies employing directional solidification have demonstrated that higher cooling rates lead to refined α-Al cells, establishing a foundation for subsequent mechanical response comprehension [14]. Studies imposing rapid solidification, such as laser powder bed fusion (LPBF), demonstrated that high cooling rates promote a dense distribution of nanoscale Al_3_(Sc,Zr) dispersoids, which markedly improve yield strength and delay recrystallization during post-processing operation [12,15]. Conversely, slower cooling in casting routes can result in coarser precipitates and localized segregation, making the microstructure more sensitive to subsequent heat treatment [16].

Zr and Sc are widely used alloying additions because they form precipitates with very similar characteristics. In particular, both elements can promote the formation of fine, coherent dispersoids/precipitates (e.g., Al_3_Sc and Al_3_Zr), which are thermally stable. Yu et al. [17] established that the mechanical strength of Al–Mg–Si–Sc–Zr alloys derives directly from the density and coherency of Al_3_(Sc,Zr) nanoparticles, which can be optimized through heat treatment schedules. Cao et al. [18] further clarified that overaging can degrade tensile performance by transforming coherent precipitates into incoherent ones, highlighting the necessity of precise thermal management. These works collectively underscore the need to understand both solidification-stage cooling and post-solid-state aging stage as interdependent parameters in microstructural design.

Despite the development of Al-Mg-Sc alloys, several aspects remain under active investigation by research groups working in this field, especially for alloys in as-cast condition. These include (1) the influence of solidification cooling rate on microstructure formation during solidification; (2) the identification of predominant phases and morphologies under a variety of solidification conditions; (3) the determination of optimal precipitation conditions during direct cooling from liquid until room temperature; and (4) influence of microstructural features, arising from both solidification and precipitation stages, on the mechanical behavior, with emphasis on their correlation to the underlying strengthening mechanisms.

In as-cast binary Al-Sc alloys, the main hardening mechanisms include grain refinement, dispersion strengthening by primary Al_3_Sc particles, and solid solution strengthening [19]. Grain refinement occurs due to the potent nucleating effect of Sc on α-Al during solidification, resulting in finer grains and improved strength. Primary Al_3_Sc particles, which form when Sc content exceeds its eutectic point, are typically faceted and semi-coherent or incoherent, acting as barriers to dislocation motion. A smaller fraction of Sc remains in solid solution, contributing to lattice distortion and solid solution strengthening. These combined effects can provide notable strength improvements in the as-cast state [20].

The innovation of the present investigation lies in its comprehensive approach to understanding the evolution of microstructure and mechanical properties in Al–Mg–Sc alloys under multiple cooling conditions, related to both solidification and subsequent solid-state cooling. Through controlled directional solidification and post-solidification cooling of varying severities, this work elucidates how thermal history governs the formation and distribution of Al_3_Sc precipitates. Although Al–Mg–Sc alloys are conventionally processed in the wrought condition, establishing a detailed baseline for the as-cast Al-5Mg-0.4Sc alloy is essential to decouple the intrinsic solidification and subsequent aging effects from those induced by deformation. This dual-stage thermal analysis not only clarifies the interplay between microsegregation, dendritic morphology, and Al_3_Sc precipitation but also provides a direct pathway for tailoring strength and ductility via aging treatments. The findings are particularly relevant for near-net-shape and additive manufacturing applications, where post-solidification cooling and aging become key tools to engineer microstructure and performance without mechanical forming.

Using the transient directional solidification technique, it will be possible to obtain the Al-5Mg-0.4Sc alloy samples with markedly different solidification rates, which will be evaluated in this work. To complement the assessment of Sc content, some samples with lower Sc levels will be used for comparative purposes, particularly regarding mechanical properties. Based on the existing knowledge for Al–Sc alloys, the present investigation aims to advance the understanding of the solidification behavior of the Al–5Mg–0.4Sc alloy. The formed phases will be identified and analyzed through optical microscopy, XRD, SEM, and thermodynamic calculations, as well as characterized with respect to their formation. Advanced TEM techniques will be employed to identify and analyze the nanometric Al_3_Sc precipitates, establishing correlations between their features and the tensile properties, as well as the serrated flow behavior observed in the stress–strain curves due to the PLC effect typical of these alloys.

Therefore, the goal of the present research was to investigate the influence of solidification and post-solidification cooling rates on the microstructural evolution, Al_3_Sc precipitation behavior, and tensile properties of an as-cast Al–5Mg–0.4Sc alloy produced by transient directional solidification.

## 2. Materials and Methods

The experimental methodology consists of three main stages: alloy fabrication via directional solidification, sample extraction, and subsequent microstructural and mechanical characterization. Directional solidification was employed to produce ingots with spatially varying cooling rates, enabling the investigation of solidification dynamics and their influence on microstructure and mechanical properties. All analyses were conducted on Al–5 wt.% Mg–0.4 wt.% Sc alloy samples extracted from distinct positions along the ingot length (from the bottom to the top).

### 2.1. Thermodynamic Calculations

To model the solidification behavior under near- and far-from-equilibrium conditions, thermodynamic simulations were performed using the CALPHAD approach. Thermo-Calc^®^ software (version 2020, Thermo-Calc Software AB, Stockholm, Sweden) and the TCAL7.1 database (2022) were utilized to predict phase transformation temperatures, reaction sequences, and equilibrium phase fractions during solidification. The alloy composition was specifically designed to promote the formation of Al_3_Sc precipitates for enhanced mechanical strength, while maintaining sufficient Mg content to facilitate solid-solution and work-hardening effects. The phase stability and solidification pathway were analyzed to help analyze appropriate control of intermetallic formation under varying cooling rates. To ensure process-relevant predictions, the CALPHAD results also considered the effects of typical impurity levels of Fe and Si, common contaminants in commercial aluminum alloys.

### 2.2. Transient Directional Solidification

The Al-5 wt.% Mg-0.4 wt.% Sc alloy was prepared using commercially pure materials: Al (99.8 wt.%), Mg (99.9 wt.%), and a master alloy of Al-2 wt.% Sc. The raw materials were cut using a band saw and weighed with a precision of ±0.01 g. Melting was conducted in an Inductotherm VIP Power-Trak induction furnace (50 kW, 3.2 kHz, Rancocas, NJ, USA). Directional solidification was carried out in a cylindrical AISI 310 stainless-steel mold (60 mm in diameter × 80 mm in length) under transient heat flow conditions. The base plate, made of water-cooled SAE 1020 steel, served as the primary heat extraction source, promoting unidirectional upward solidification. To minimize radial heat losses and ensure thermal directionality, the inner mold walls were coated with a silicon-alumina ceramic layer. Water cooling was carried out until room temperature was reached.

Six K-type thermocouples (ANSI C96-1963), housed in AISI 304 stainless-steel sheaths, were positioned in 1.5 mm-diameter holes along the mold wall to monitor temperature (T) as a function of time (t) at discrete positions. According to the standard specification, the thermocouples present a typical accuracy of ±2.2 °C or ±0.75% of the measured temperature. Temperature data were acquired using a Lynx ADS-1800 system (Lynx Tecnologia Eletrônica Ltd., São Paulo, Brazil) at a sampling rate of 5 Hz per channel. Cooling rates (Ṫ) and solidification velocities (V) were determined from the T–t profiles based on the position of the liquidus, following well-established procedures [21,22,23,24,25].

The cooling rates covered a range from 1.3 °C/s to 15.3 °C/s along the six positions in the ingot (5 mm, 10 mm, 15 mm, 20 mm, 25 mm and 44 mm). Samples from six distinct positions were extracted for microstructural and mechanical analyses.

**Figure 1:**
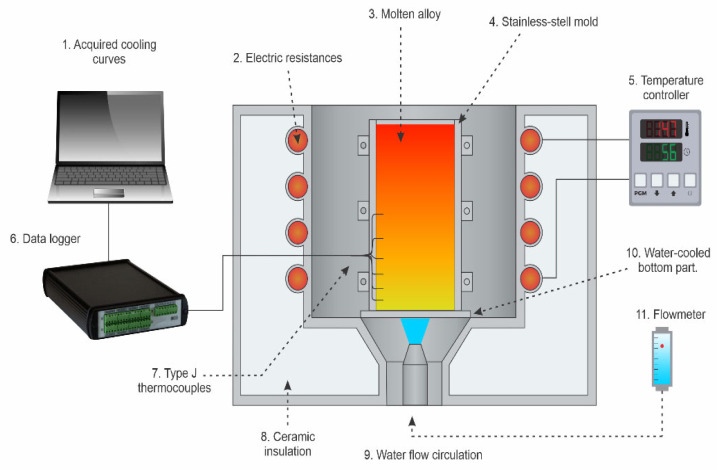
Representation of the upward directional solidification device.

Figure 1 shows the upward, water-cooled directional solidification apparatus employed to cast the alloy.

### 2.3. Microstructural Characterization

Microstructures were characterized using optical microscopy (Olympus BX41M-LED, Tokyo, Japan) and SEM (XL-30 FEG, Philips, Amsterdam, The Netherlands) with energy-dispersive spectroscopy (EDS, Xplore 30, Oxford Instruments, Peabody, MA, USA). Etching for microstructural observation was performed using a solution of equal parts HCl, HNO_3_, and H_2_O, with 1/30 HF added, applied with a cotton swab for 5 s.

X-ray diffraction (XRD) was performed to support microstructural phase identification on samples solidified at representative cooling rates (e.g., 15.3 and 1.3 °C/s). Measurements were carried out with a Bruker D8 Advance ECO diffractometer (Bruker AXS, Karlsruhe, Germany) using Cu Kα radiation (λ = 1.5418 Å). Data were collected over a 2θ range of 5–90° with a step size of 0.02°. Two very different cooling rates have been chosen intentionally to bracket the solidification window relevant to casting and to probe two distinct microstructural regimes. The lower cooling rate represents a coarsening regime typical of bulk sections, whereas the higher rate represents a higher-undercooling, diffusion-limited regime typical of faster heat extraction. This strategy maximizes the contrast in microstructure morphology, demonstrating that the reported trends are robust rather than case-specific.

X-ray fluorescence (XRF) analyses were carried out with the aim of identifying the variation in the elements forming the alloy along the length of the casting. The equipment used was a Shimadzu, EDX-720 model (Shimadzu, Kyoto, Japan).

To gain further insight into microstructural features, advanced transmission electron microscopy (TEM) techniques were employed. High-resolution imaging and chemical analysis were carried out using a ThermoFisher Talos F200X G2 (Thermo Fisher Scientific, Waltham, MA, USA), a 200 kV field-emission gun (FEG) scanning transmission electron microscope (STEM) equipped with a Super-X energy-dispersive spectroscopy (EDS) system.

Specimen preparation for TEM involved initial mechanical thinning to ~50 μm, followed by punching 3 mm discs. Final thinning and surface polishing were completed using a Gatan Dimpler and the Gatan Precision Ion Polishing System (PIPS, Gatan Inc., Pleasanton, CA, USA) to achieve electron transparency suitable for high-resolution analysis.

### 2.4. Mechanical Testing

Tensile mechanical behavior was evaluated using dogbone-shaped specimens machined from transverse sections of the directionally solidified ingot, in accordance with ASTM E8/E8M-22 [26]. The specimen axis was aligned parallel to the growth direction. The specimens had a gauge length of 30 mm, a width of 4 mm, and a thickness of 2 mm. Tensile tests were performed at a constant strain rate of 3.0 × 10^−3^ s^−1^ using an Instron 5500R universal testing machine (Instron, Norwood, MA, USA) equipped with a 5000 N load cell and a calibrated video extensometer. Tests were conducted at selected positions along the ingot corresponding to cooling rates of 1.6, 2.3, 3.9, and 11.2 °C/s, covering a wide solidification spectrum. Although these cooling rates differ slightly from those used for microstructural characterization, they represent closely comparable solidification conditions. Each testing condition was evaluated in triplicate.

## 3. Results and Discussion

### 3.1. Solidification

**Figure 2:**
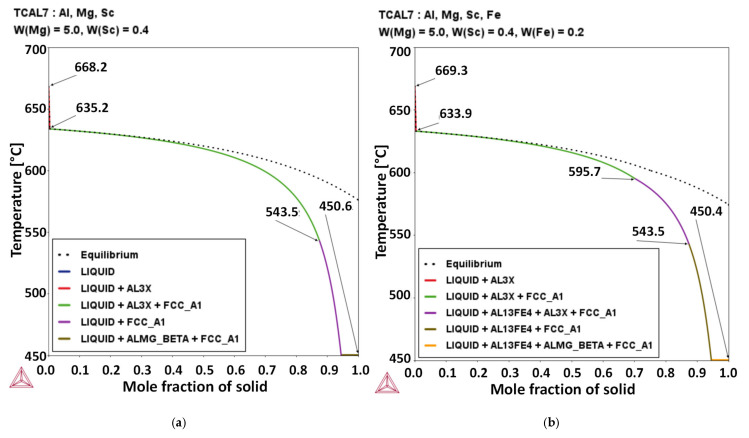
Equilibrium and Scheil solidification paths calculated for the Al-5Mg-0.4Sc alloy: (**a**) without and (**b**) with Fe addition.

The CALPHAD method was used to predict the phase evolution during the solidification of the Al–5Mg–0.4Sc alloy. Figure 2a shows the calculated solidification path, where solidification begins at approximately 668 °C, and with the formation of the primary FCC_A1 phase (α-Al matrix) starting at approximately 635 °C. It is worth noting that a secondary phase (AL3X, likely Al_3_Sc) forms alongside FCC_A1. Near the end of solidification, at approximately 450.6 °C according to the Scheil profile, a Mg-rich intermetallic phase (ALMG_BETA) appears in coexistence with the α-Al, forming the eutectic constituent. This sequence highlights key transformation temperatures and the progressive formation of solid phases, providing valuable insight into the microstructural evolution of the alloy during cooling from the liquid.

In Figure 2b, the comparison between the CALPHAD-predicted solidification paths of the Al–5Mg–0.4Sc alloy with 0.2 wt.% Fe reveals that such Fe content at levels typically present as impurities in secondary sources can significantly alter the phase formation during solidification. Considering the alloy with 0.2%Fe, solidification begins at 669.26 °C. Subsequently, precipitation of an Al_3_Sc-type phase (AL3X) occurs and continues until 543.52 °C. The onset of FCC_A1 phase formation is observed at 633.91 °C, and solidification ends with the formation of a Mg-rich phase (ALMG_BETA) at 450.44 °C. It is important to note that the formation of a Fe-rich intermetallic phase (AL13Fe4) occurs at around 595.7 °C, which alters the phase sequence and coexists with FCC_A1 and AL3X before the final eutectic. The appearance of AL13Fe4 when Fe is considered indicates that the Fe solubility limit is exceeded. Therefore, Fe content plays a critical role in microstructural evolution and must be considered here for the investigation of the phases formed.

**Figure 3:**
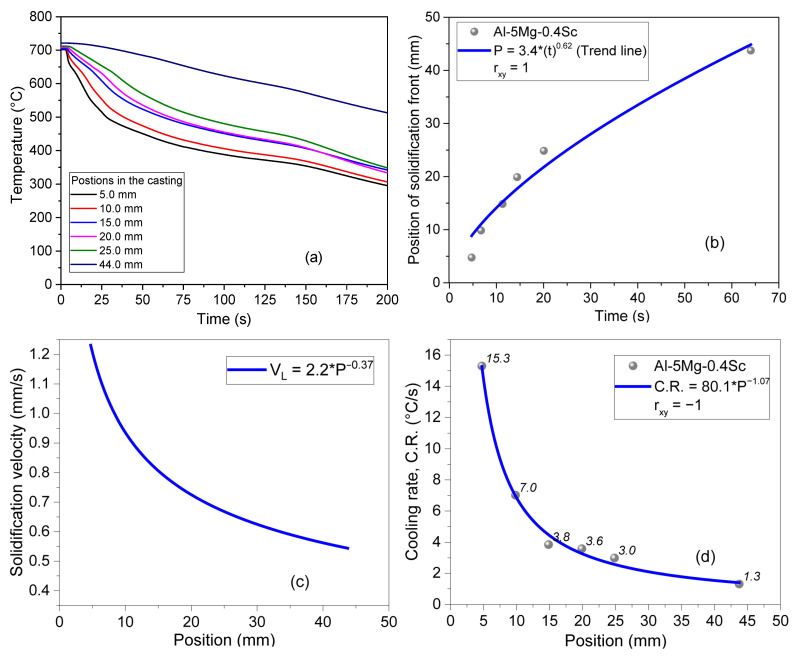
(**a**) Temperature x time profiles; (**b**) displacement of the solidification front with time; (**c**) solidification velocity; and (**d**) cooling rate during solidification of the Al-5Mg-0.4Sc alloy. r_xy_ is the Pearson correlation coefficient.

Figure 3 shows the thermal data evolution and solidification behavior of the Al–5Mg–0.4Sc alloy during unidirectional solidification. In Figure 3a, the temperature profiles recorded at increasing positions, P, from the heat-extracting surface reveal faster cooling near the base and progressively slower cooling at more distant positions. For instance, at 5 mm from the water-cooled base, the cooling rate reaches 15.3 °C/s, decreasing to 7.0 °C/s at 10 mm, 3.8 °C/s at 15 mm, and 3.6 °C/s at 20 mm. Beyond this, cooling rates continue to decline, with values of 3.0 °C/s at 25 mm, 1.3 °C/s at 44 mm. These values demonstrate the steep thermal gradient imposed during directional solidification. Figure 3b depicts the evolution of the solidification front based on the liquidus displacement (P) over time (t), which follows a power-law relationship described by P = 3.4·t^0.62^, indicating a progressive reduction in front velocity over time. In Figure 3c, the solidification velocity is shown to decrease with distance from the chill surface, following V_L_ = 2.2·P^−0.37^. This behavior reflects the decreasing heat extraction rate along the casting length [27,28,29,30,31]. Finally, Figure 3d consolidates the cooling rate (C.R.) trend, confirming a strong inverse dependence on relative position, expressed by CR = 80.1·P^–1.07^, with excellent correlation to experimental data (r_xy_ = −1). This study of solidification kinetics is very important because it allows for the extraction of samples with microstructural formation under different kinetic conditions, which can affect the phases formed, their morphology, solute saturation, as well as the size and distribution of these phases.

**Figure 4:**
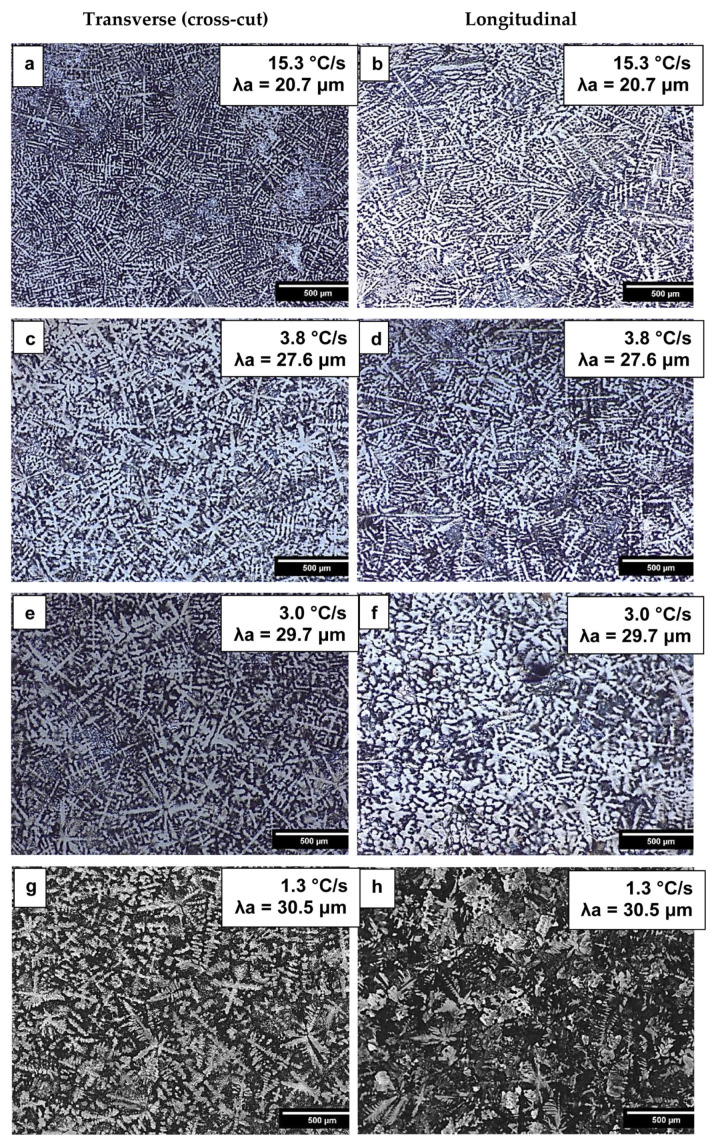
Longitudinal and transverse optical microstructure of Al-5Mg-0.4Sc alloy for samples at 15.3 °C/s (**a**,**b**), 3.8 °C/s (**c**,**d**), 3.0 °C/s (**e**,**f**) and 1.3 °C/s (**g**,**h**).

The optical microstructures obtained from both longitudinal and transverse sections are shown in Figure 4. The bright areas in Figure 4 represent the α-Al dendrites, while the dark areas correspond to the other formed phases, mainly Al_3_Sc, AlFe, and β-Al_3_Mg_2_, as will be further characterized later in this paper. Fe-rich intermetallics are hereafter referred to generically as Al–Fe phases, encompassing both stable and metastable Al–Fe compounds reported in the specialized literature. Although DS typically leads to the development of columnar grain structures, the Al-5Mg-0.4Sc alloy investigated in this work displays a fully dendritic equiaxed morphology, as observed in Figure 4. This behavior is attributed to the presence of scandium (Sc), which is widely recognized for its potent grain-refining effect in aluminum alloys. The addition of Sc alters the solidification dynamics by promoting heterogeneous nucleation and inhibiting columnar growth, thereby influencing the resulting grain structure.

Similar equiaxed morphologies have been reported in previous studies on Al-Mg-Sc alloys with varying Sc contents processed under conventional casting conditions [32,33]. However, it has been demonstrated that when the Al-5Mg alloy containing only 0.1 wt.% Sc was subjected to directional solidification under similar thermal conditions, a predominantly columnar grain structure was formed [14] underscoring the critical role of Sc concentration in determining the final grain morphology.

The addition of 0.4 wt.% Sc was sufficient to suppress columnar grain formation, promoting the development of equiaxed grains in both the longitudinal and transverse solidification directions in Figure 4. In the corresponding micrographs, the dendritic arms exhibit short inter-arm distances. To account for the full range of measured values, the average dendritic arm spacing (λa) is indicated in Figure 4. As expected, λa decreases inversely with increasing cooling rate. The modification of the dendritic spacing was more pronounced in the regions subjected to higher cooling rates, that is, those closer to the metal/mold interface. This refinement near the metal/mold interface occurs because higher cooling rates reduce the time available for solute diffusion and dendrite coarsening, leading to finer microstructural scales.

**Figure 5:**
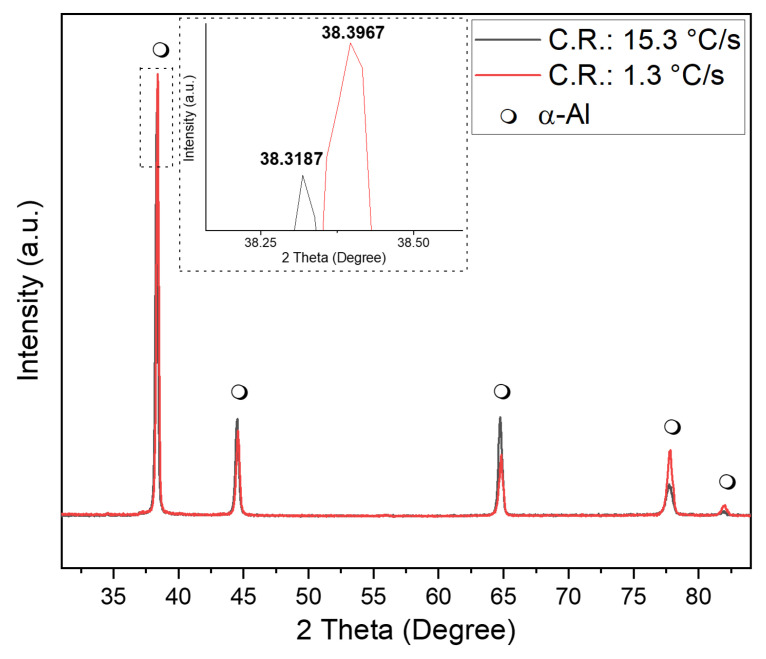
X-ray diffraction (XRD) patterns for the Al-5Mg-0.4Sc alloy samples solidified at different cooling rates: 15.3 °C/s (black line) and 1.3 °C/s (red line). All detected peaks correspond to the face-centered cubic (FCC) α-Al phase, as indicated by the black markers (μ), with no evidence of secondary phase formation within the detection limits of the measurements.

As evidenced by the XRD diffraction patterns in Figure 5, the Al-5Mg-0.4Sc alloy exhibits distinct crystallographic responses under different cooling rates. The inset highlights the main diffraction peak near 2θ ≈ 38.3°, where a clear shift is observed between the two cooling conditions. The sample solidified at the higher cooling rate (15.3 °C/s) shows a peak at 38.3187°, while the slower-cooled sample (1.3 °C/s) presents the same peak at a slightly higher angle of 38.3967°. This shift to higher 2θ values at lower cooling rates suggests subtle changes in the α-Al lattice parameters, potentially due to microsegregation effects or solute redistribution during slower solidification.

These findings indicate that while both cooling conditions result in XRD patterns dominated by the α-Al matrix, although additional secondary phases are present at the microstructural level, the cooling rate influences lattice distortion, likely due to variations in solute trapping and solid solution supersaturation. Faster cooling can promote greater solute retention in the matrix, slightly expanding the lattice and causing the diffraction peaks to shift toward lower angles [34].

### 3.2. Advanced Microstructural Characterization Based on SEM and TEM

**Figure 6:**
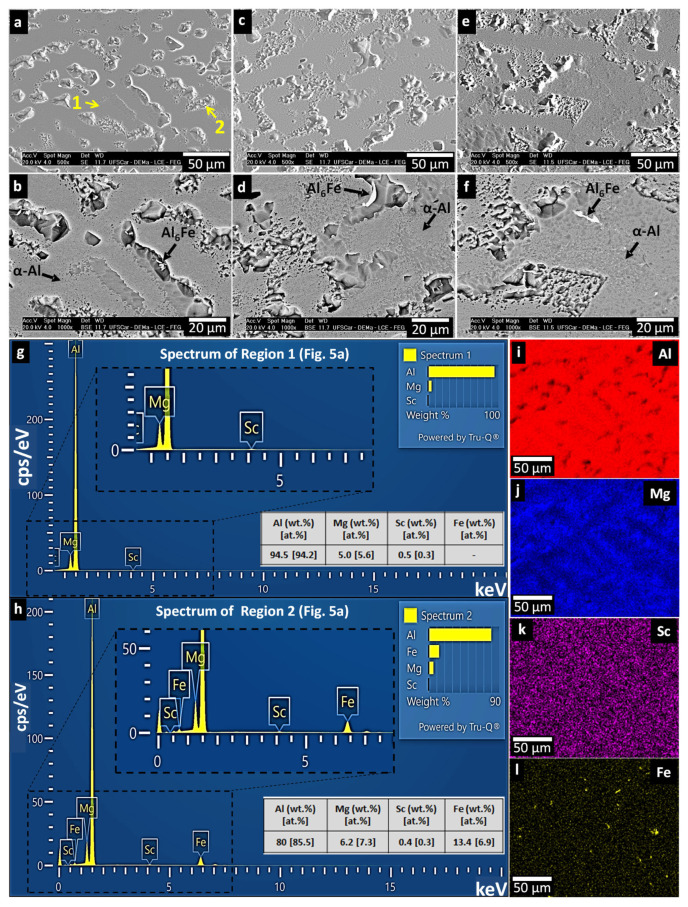
SEM micrographs (SE/BSE modes) illustrating the microstructural constituents formed in the Al-5Mg-0.4Sc alloy at different cooling rates: (**a**,**b**) 15.3 °C/s; (**c**,**d**) 3.8 °C/s; and (**e**,**f**) 3.0 °C/s. (**g**,**h**) EDS point analysis corresponding to Regions 1 and 2 indicated in (**a**), including the summarized elemental compositions in weight and atomic percent; (**i**–**l**) EDS elemental maps.

Figure 6 shows SEM images in high magnification detail, corresponding to the same cooling rates as in Figure 4. The aim is to more carefully analyze the interdendritic phases, focusing on those with sizes large enough to be detected by SEM. A micromorphological transition was observed along the length of the sample. Near the bottom region, where the cooling rate was 15.3 °C/s, the microstructure appeared more refined. As the distance from the base increased, the cooling rate decreased to around 3.0 °C/s, resulting in progressively coarser structures. However, SEM (SE/BSE) analyses revealed no substantial variation in the types of micrometric phases formed across these regions, as shown in Figure 6. The BSE images confirm the presence of the AlFe intermetallic phase, typically associated with Fe contamination from commercial-grade aluminum. Although equilibrium phase diagram calculations (Figure 2) predict the formation of the stable Al_13_Fe_4_ phase, the phase observed in the present work corresponds instead to the metastable Al_6_Fe compound. The occurrence of Al_6_Fe under such conditions is well documented in the literature [35,36,37], as this metastable phase preferentially forms during non-equilibrium solidification of Al–Fe alloys. This behavior is consistent with the conditions inherent to directional solidification, where spatial variations in cooling rate promote the formation and growth of metastable intermetallics rather than the equilibrium phases predicted by CALPHAD. This phase is known to degrade mechanical properties, although in certain alloy systems, its impact can be mitigated through compositional adjustments [38,39].

**Figure 7:**
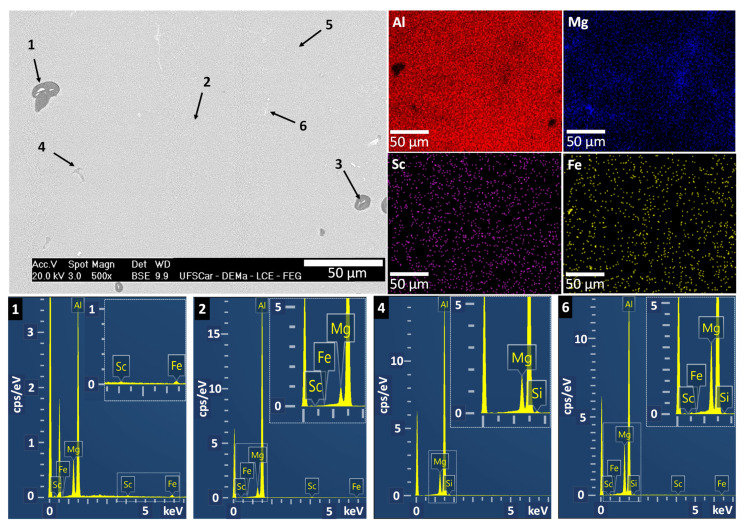
SEM-EDS micrographs and some of the representative EDS counts of the phases formed in the Al-5Mg-0.4Sc alloy at 1.3 °C/s. Representative points: Point #1—Al_3_Mg_2_, Point #2—matrix, Point #4—MgSi, Point #6—Al_3_Mg_2_.

**Figure 8:**
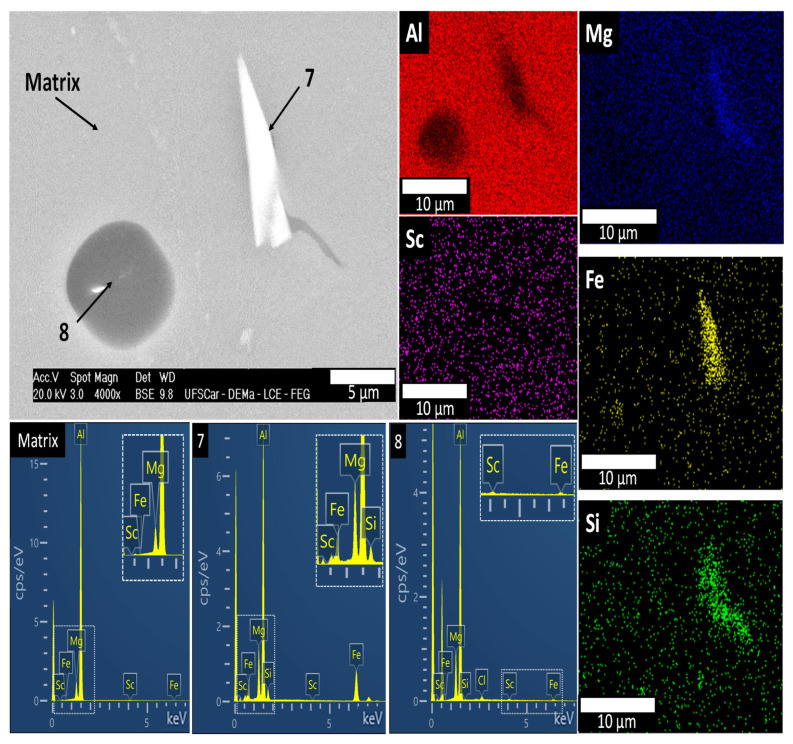
SEM-EDS images and EDS point data of the phases formed in the Al-5Mg-0.4Sc alloy at 1.3 °C/s.

The presence of the α-Al matrix in the microstructure is unequivocal. However, two additional phases predicted by thermodynamic simulations (β-Al_3_Mg_2_ and Al_3_Sc) were not directly identified through SEM analysis. Figure 7 shows EDS results (both point and elemental mapping), which confirm the presence of the AlFe intermetallic and the Mg-enriched phases. All the EDS points in Figure 7 and Figure 8 are summarized in Table 1. The retention of the β-Al_3_Mg_2_ phase during one of the sample preparation methods employed here has been facilitated by the absence of chemical etching during sample preparation, which involved vibratory polishing with silica-based media for 24 h, whose results can be seen in Figure 7. In this context, EDS points #1, #5, and #6 reveal a strong interaction between Al and Mg and indicate the formation of the Al_3_Mg_2_ phase, as described in Table 1. Moreover, relatively high Fe content in the region corresponding to the point #1 in Table 1 can indicate the local formation of Fe-containing phases. Furthermore, the straight-edged voids observed in the SEM images in Figure 6 are characteristic of β-Al_3_Mg_2_ dissolution during sample preparation, strongly indicating its original presence prior to etching or polishing.

**Figure 9:**
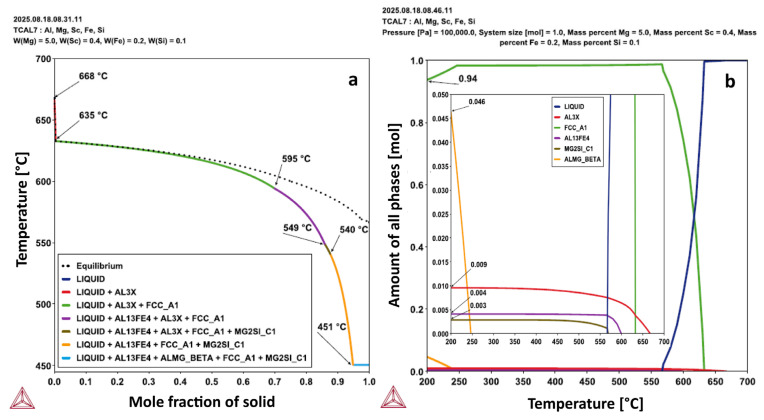
(**a**) CALPHAD diagrams computing the solidification path for the Al-5Mg-0.4Sc alloy considering contamination with Si and Fe; and (**b**) Evolution of the molar fractions of the phases as a function of temperature for the Al-5Mg-0.4Sc alloy from the liquid state considering contamination with Si and Fe.

Figure 8 shows a higher-magnification SEM image along with the EDS elemental mapping, including typical the signal count spectra, where Si was detected within the same Mg-rich region via both point analysis and mapping, as indicated through point #7 in Table 1. The presence of Si likely originates from the secondary Al ingot used in alloy production, which contains approximately 0.1 wt.% Si, as measured through XRF. Additional thermodynamic calculations in Figure 9 have been performed incorporating the Si trace content into the alloy composition. These calculations suggest the potential formation of the Mg_2_Si phase (MG2SI_C1) in a very low volume fraction, approximately 0.3%. Moreover, according to the Scheil simulation results shown in Figure 9a, the alloy containing trace amounts of Si and Fe tends to form a quaternary a-Al+AlFe+AlMg+Mg_2_Si eutectic, which was partially identified at points #7 and #8 in Figure 8, comprising the AlFe, Mg_2_Si, and AlMg phases. In other words, the CALPHAD results have been consistent with the EDS findings. It is worth noting, at this point, that the identification of these phases refers to their primary formation during solidification.

To further characterize the microstructure of the Al-Mg-Sc alloy, TEM analyses were conducted on samples corresponding to the lowest and highest solidification rates in this study, namely 1.3 °C/s and 15.3 °C/s, which are associated with the first and sixth thermocouples, respectively, in the as-solidified body. In the following, emphasis is placed on identifying nanometric Al_3_Sc precipitates formed during cooling-down after solidification stage and examining the characteristics of the Al-rich matrix.

**Figure 10:**
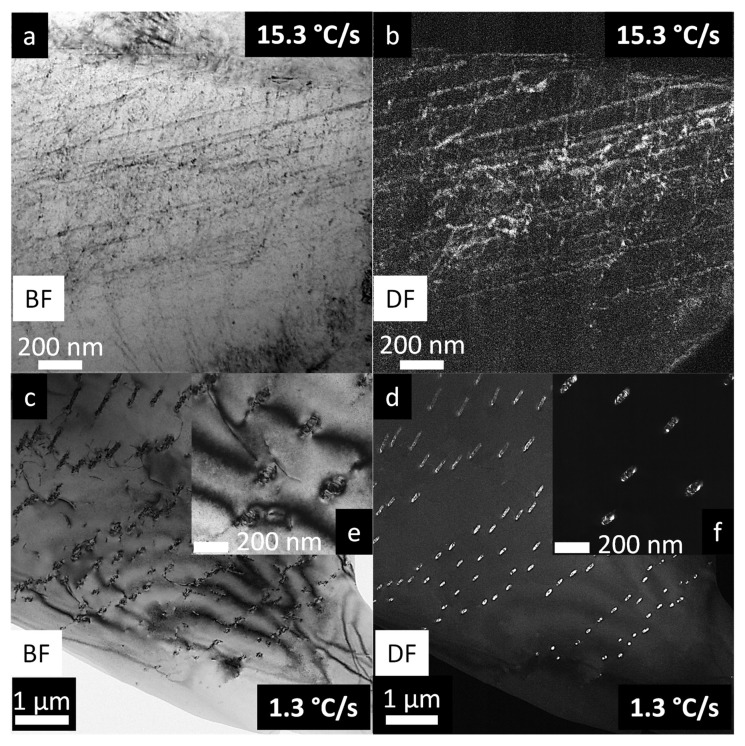
TEM micrographs of the directionally solidified Al–5Mg–0.4Sc alloy: (**a**,**b**) bright- and dark-field images of the 15.3 °C/s sample; (**c**,**d**) bright- and dark-field images of the 1.3 °C/s sample; (**e**,**f**) corresponding detailed views of (**c**,**d**).

Figure 10 shows conventional TEM bright-field (BF) and dark-field (DF) images related to both samples solidified at high and low rates. In Figure 10a (BF) and 10b (DF), associated with the higher cooling rate (15.3 °C/s), the α-Al matrix as well the presence dislocations are clearly visible. In the case of low-cooling rate samples, Figure 10c (BF) and 10d (DF) reveal a distinct microstructure, with oriented rod-like precipitates in the matrix and dislocations cutting through them. Figure 10e,f are magnified images of Figure 10c (BF) and 10d (DF) to more clearly reveal microstructural details of the matrix, dislocations and precipitates. The precipitates formed near the top of the directionally solidified ingot exhibit an aspect ratio of around 2:1 to 4:1, with lengths of 100–200 nm and widths of around 50 nm. Under faster solidification conditions (15.3 °C/s), the formation and growth of the Al_3_Sc nanoprecipitates in an Mg-containing Al matrix are likely to be significantly hindered due to the limited time available for diffusion and the potential solute-drag effect of Mg. In contrast, the much longer solidification times in slowly solidified samples (1.3 °C/s) facilitate Sc diffusion and, consequently, the precipitation and growth of Al_3_Sc, as evidenced in Figure 10c–d.

In the BF images of the samples solidified at the lowest rate (1.3 °C/s), Figure 10c,e, the pronounced interactions between dislocations and precipitates are observed. These interactions manifest as (i) dislocations bowing around the precipitates (Orowan looping) or (ii) dislocations shearing partially or fully incoherent precipitates, as suggested by the characteristic strain-contrast fields around these particles [40]. This loss of coherency has also been reported by Marquis and Seidman [8], who identified interfacial dislocations once Al_3_Sc precipitates reached approximately 40 nm in a binary Al–0.3Sc alloy. The distinct microstructural features—namely, a homogeneous solid solution at 15.3 °C/s and incoherent precipitates at 1.3 °C/s—are consistent with the cooling-rate dependence of phase stability in this system and have direct implications for mechanical properties, as discussed later.

**Figure 11:**
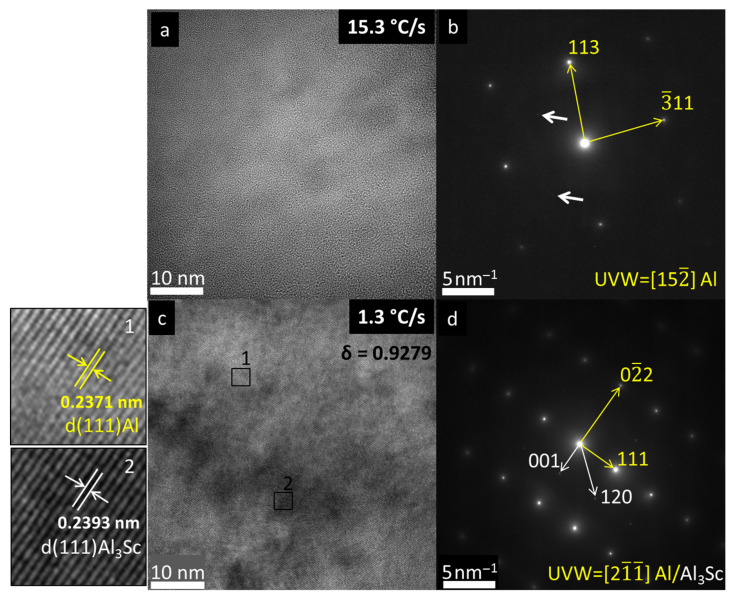
(**a**) HRTEM and (**b**) associated SAD related to the sample at 15.3 °C/s; (**c**) HRTEM and (**d**) associated SAD related to the sample at 1.3 °C/s of the Al-5Mg-0.4Sc alloy. Yellow arrows indicate α-Al spots, and white arrows indicate Al_3_Sc spots. On the left side of the HRTEM images, selected regions from Figure 11c are displayed, where interplanar distances were measured: region #1 corresponding to the α-Al and region #2 to the Al_3_Sc.

The BF/DF TEM images in Figure 10 were acquired under conventional imaging conditions, without alignment to a specific crystallographic zone axis, as the objective was to evaluate general microstructural features (matrix contrast, dislocation structures, and Al_3_Sc precipitate morphology) rather than to extract crystallographic information. For deeper insight into the microstructure under both solidification conditions, advanced TEM techniques—including HRTEM, SAED, and STEM-EDS mapping—were subsequently employed, as present in Figure 11, where the incident beam direction [uvw] is explicitly provided.

Figure 11 presents HRTEM images and their corresponding selected-area diffraction (SAD) patterns. The HRTEM images under the distinct solidification conditions (Figure 11a,c) highlight differences in the microstructural homogeneity, evidenced by phase-contrast variations: subtle at the highest cooling rate (15.3 °C/s) and pronounced at the lowest rate (1.3 °C/s). Interplanar distances (d) measured via ImageJ [41] in Figure 11c (1.3 °C/s) ranged from 0.237 nm to 0.239 nm, revealing clear phase contrast between the α-Al matrix and Al_3_Sc precipitates. The measured value of d_α-Al_ was equal to 0.2371 nm and *d_Al_3_Sc_* was 0.2393 nm. Such values are close to the theoretical value of the interplanar distance d(111)_Al_ = 0.234 nm. The misfit between the two lattices (δ) is defined by [42] according to Equation (1),

(1) $$\delta =\frac{d_{Al_{3}Sc}-{\;d}_{Al}}{d_{Al}}$$

resulting in 0.928 of misfit between α-Al and Al_3_Sc phases and was indicated on Figure 11c. Both phases are cubic (α-Al: Fm-3m, A1, a = 4.0496 Å; Al_3_Sc: Pm-3m, L1_2_, a = 4.103 Å [20]), which explains their similar d-spacing. The SAD patterns further corroborate these microstructural differences, since Figure 11b (15.3 °C/s) shows dominant α-Al spots with only two faint, discernible Al_3_Sc (100) reflections (indicated by white arrows on Figure 11b), while Figure 11d (1.3 °C/s) exhibits more intense Al_3_Sc spots (white arrows) alongside the α-Al reflections (yellow arrows). These fainter spots for the sample at 15.3 °C/s indicate reduced precipitation and a higher amount of Sc remaining in solid solution.

**Figure 12:**
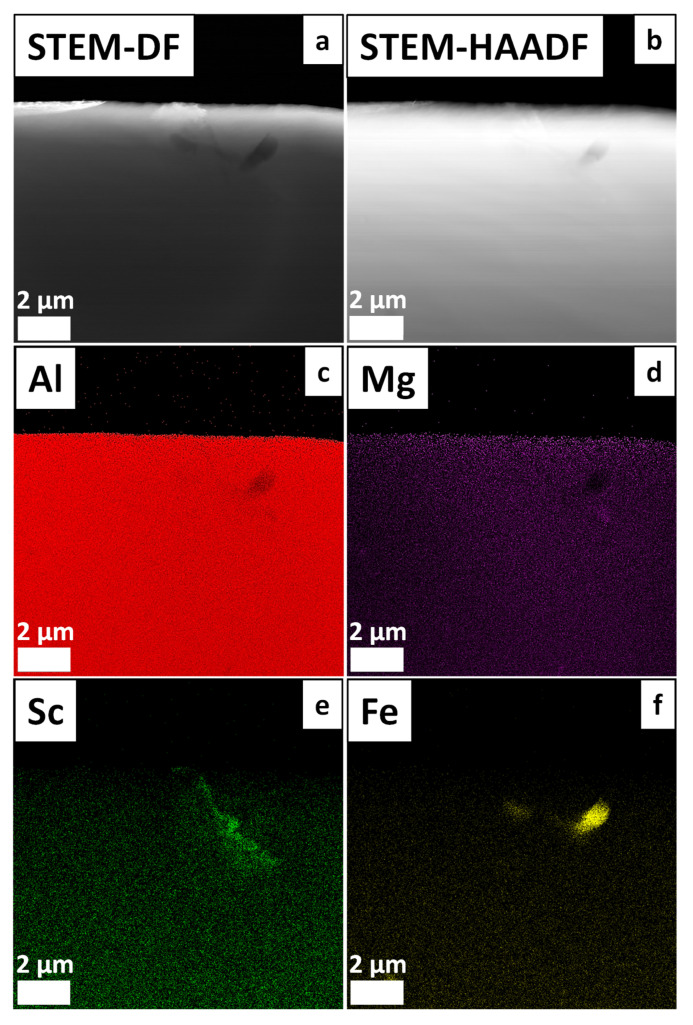
STEM analysis of the Al-5Mg-0.4Sc alloy solidified at 15.3 °C/s: (**a**) STEM dark-field (DF) image; (**b**) STEM-HAADF image; (**c**–**f**) and corresponding EDS elemental maps of Al, Mg, Sc and Fe.

Figure 12 shows the α-Al grains at the top of the STEM-DF image as well as elemental distribution in the Al matrix and around the grain boundaries in the sample at 15.3 °C/s. The STEM-DF (dark-field) image (Figure 12a) reveals distinct α-Al grain orientations most notably in the upper region of the image, where crystallographic contrast is pronounced. Furthermore, a submicrometric Fe-rich intermetallic phase is observed, as confirmed by the elemental mapping (Figure 12f). Despite the presence of this Fe-rich phase revealed by the EDS mapping, no deleterious effects on the mechanical properties of this specific sample have been observed, as will be discussed in Section 3.3. In contrast, the STEM-HAADF image (Figure 12b) exhibits more homogeneous contrast across the α-Al matrix grains, with no evidence of secondary phase formation at this magnification. The distribution of Sc in the sample at 15.3 °C/s reveals unexpected segregation behavior. Despite the absence of Al_3_Sc nanoprecipitates in this sample, the STEM-EDS mapping detected Sc enrichment at grain boundaries (Figure 12e). This segregation is likely associated with the dendrite boundaries, accompanied by the precipitation of Al_3_Sc particles from the liquid, as shown by the CALPHAD-predicted solidification path in Figure 2 (Section 3.1). Accordingly, segregation develops during the solidification stage and is observed here filling a region approximately 1.5–5 µm in size.

**Figure 13:**
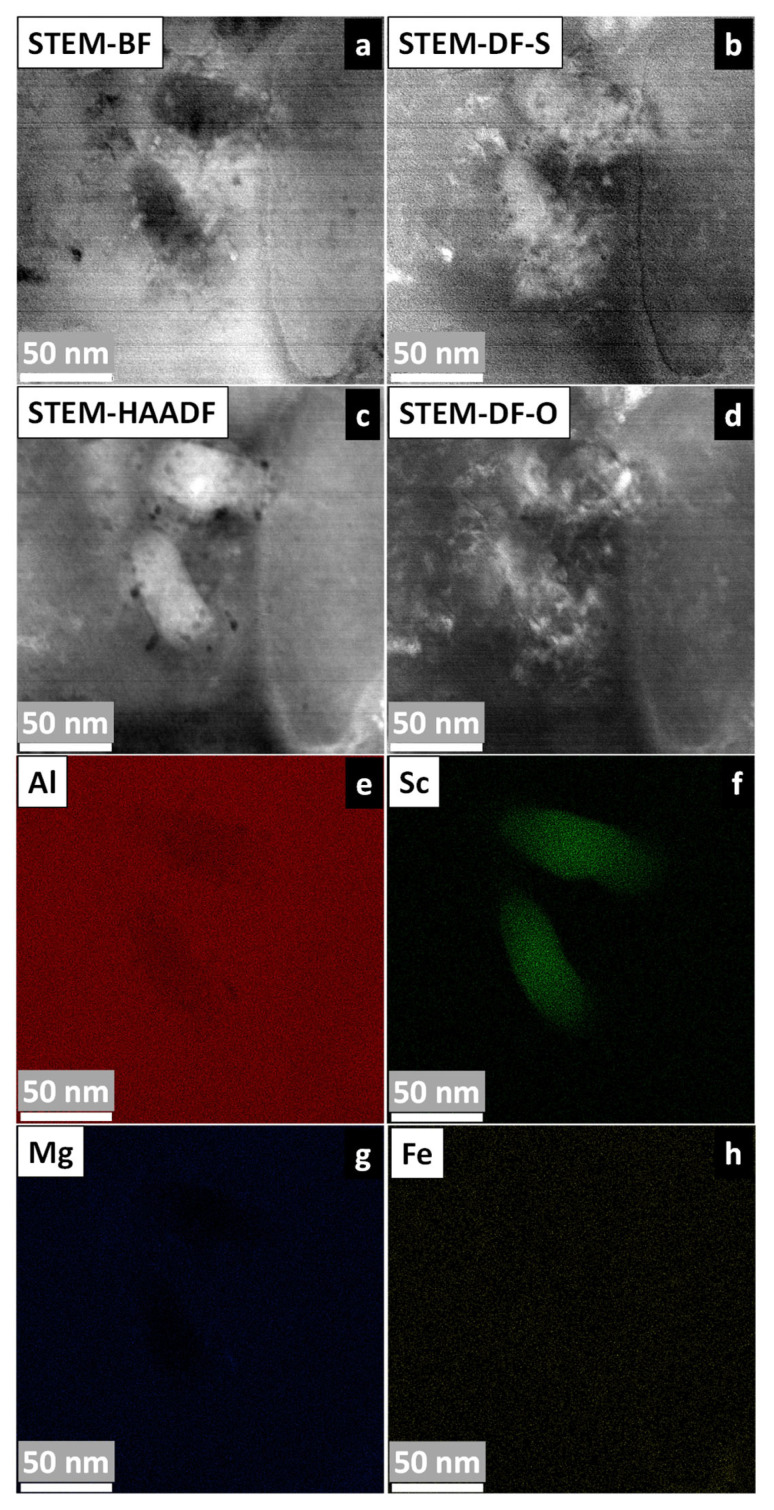
STEM-EDS analysis of Al-5Mg-0.4Sc alloy solidified at 1.3 °C/s: (**a**) STEM-BF; (**b**) STEM-DF-S; (**c**) STEM-HAADF; (**d**) STEM-DF-O images showing α-Al grains and Al_3_Sc precipitates. (**e**–**h**) And corresponding EDS maps of Al, Mg, Sc, an Fe (not detected).

For the samples as 1.3 °C/s, Figure 13 presents conclusive evidence of Al_3_Sc precipitation. In Figure 13 (a) STEM-BF, (b) STEM-DF-S, (c) STEM-HAADF, and (d) STEM-DF-O images are shown, each revealing distinct grain contrast of the α-Al matrix and Al_3_Sc precipitates through their characteristic detector-specific intensity variations. The accompanying EDS elemental maps (Figure 13e–h) confirm homogeneous Al and Mg color contrast, while Sc maps clearly delineate the precipitates’ spatial arrangement. Notably, the multi-detector approach provides complementary microstructural information, with STEM-DF-S emphasizing strain fields around precipitates and STEM-DF-O highlighting orientation-dependent contrast. This comprehensive characterization unambiguously demonstrates that slower solidification promotes Al_3_Sc formation, contrasting sharply with the grain-boundary-limited Sc distribution observed at higher cooling rates.

**Figure 14:**
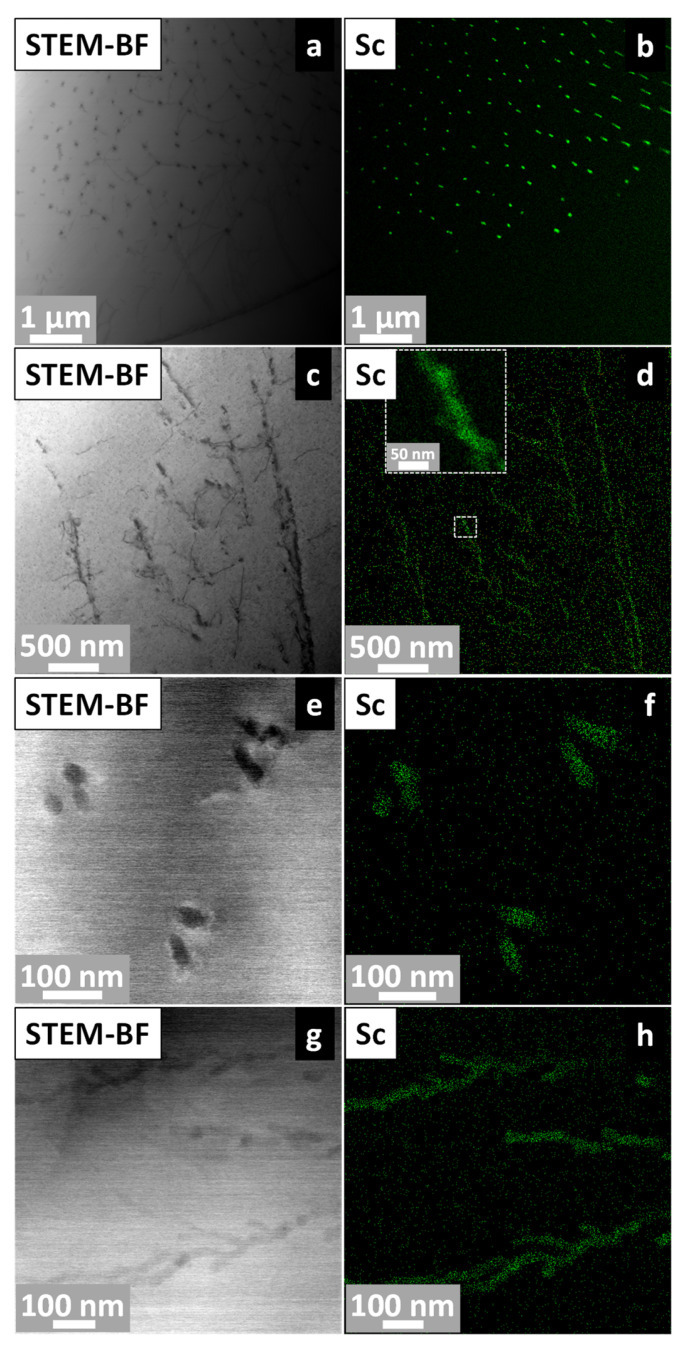
STEM-EDS analysis of the Al–5Mg–0.4Sc alloy solidified at 1.3 °C/s. (**a**,**c**,**e**,**g**) STEM-BF images showing α-Al grains and various Al_3_Sc precipitate morphologies; and (**b**,**d**,**f**,**h**) corresponding EDS-Sc maps.

Distinct types of precipitates located in different regions of the sample solidified at 1.3 °C/s were observed in the TEM analyses (Figure 14). Two predominant morphologies were identified: (i) discrete, rod-shaped nanoprecipitates (Figure 14a,b), and (ii) locally interconnected branched-like Sc-rich regions (Figure 14c,d). Although the field of view in Figure 14c,d does not allow unequivocal identification of grain boundaries, the Sc-EDS map (inset of Figure 14d, 50 nm scale bar) reveals branched and spatially extended Sc-enriched regions that are consistent with segregation pathways typically associated with boundary-like areas. Higher-magnification images (Figure 14e–h) show that the rod-like precipitates maintain well-defined interfaces with the matrix, whereas the Sc-enriched branched regions display diffuse transitions in contrast. This coexistence of localized rod-shaped precipitates and extended Sc-rich branched morphologies suggests that cooling-rate-dependent diffusion of Sc may promote different precipitation pathways during post-solidification cooling. It is important to note that the TEM bright-field and EDS images in Figure 14c,d were acquired from thin regions where diffraction contrast was relatively uniform. As a result, grain boundaries were not resolvable within this field of view, and the Sc-rich branched morphology is described based on chemical contrast rather than boundary identification.

**Figure 15:**
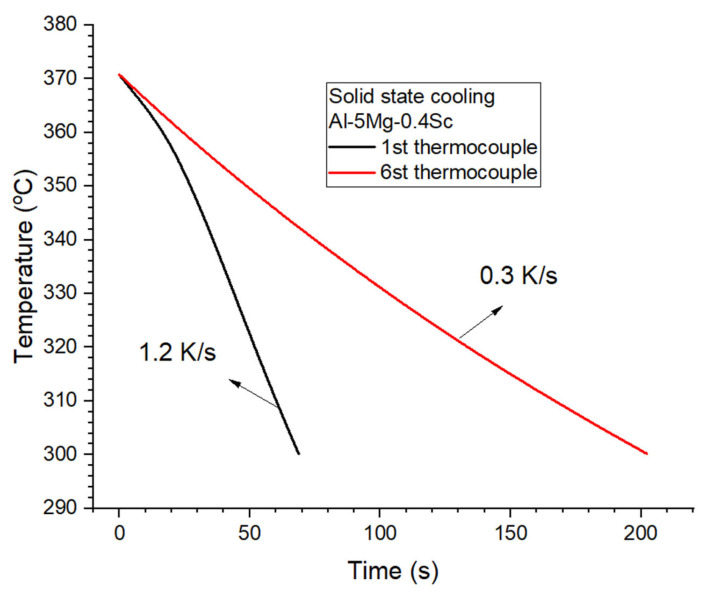
Cooling curves recorded during solid-state cooling of the directionally solidified Al–5Mg–0.4Sc alloy for the first (black) and sixth (red) thermocouples positioned along the ingot length.

The cooling rate within the temperature range of Al_3_Sc precipitation in Figure 15 regulates growth dynamics of this phase, thereby determining its final size, spatial distribution, and degree of coherency within the Al matrix. The 370–300 °C range corresponds to the active precipitation window of Al_3_Sc during solid-state cooling. Around 370 °C, CALPHAD predictions and experiments indicate the onset of precipitation, while below 300 °C scandium diffusivity in aluminum (≈10^−20^–10^−21^ m^2^ s^−1^) becomes too low to sustain further growth [9,10,13]. At the higher cooling rate (1.2 K/s, 1st thermocouple at 5 mm), the rapid temperature decreases limited scandium diffusion, retaining more Sc in solid solution after solidification. Slower cooling (0.3 K/s, 6st thermocouple at 44 mm), on the contrary, promotes coarser and less uniformly distributed precipitates, which is consistent with previous findings for Al–Sc and Al–Mg–Sc alloys [19,43]. Moreover, literature reports that Al_3_Sc nanoparticles tend to develop faceted or truncated morphologies, reflecting the influence of interfacial energy anisotropy and diffusion-controlled growth [8,43]. Under slower cooling or extended aging, these particles typically reach 20–50 nm [19].

The tendency of Al_3_Sc precipitates in Al–Mg–Sc alloys to adopt elongated rather than spherical or near-spherical morphologies has been firstly demonstrated in the studies developed by Marquis and Seidman [8,44] using homogenization followed by aging. It arises from the local crystallographic anisotropy of the L1_2_ phase and the reduction of elastic strain energy. Anisotropic interface energies and diffusion rates along different crystallographic directions promote preferential growth. In dilute Sc-containing alloys (~0.1 Sc), the small lattice misfit (~1%) keeps Al_3_Sc precipitates nearly equiaxed and faceted. At higher Sc content (~0.4 wt%) or with other phases present, elastic strain energy becomes more important than interface energy. This drives elongation along low-modulus crystallographic directions to reduce the total energy penalty.

### 3.3. Mechanical Behavior and Strengthening Mechanisms

**Figure 16:**
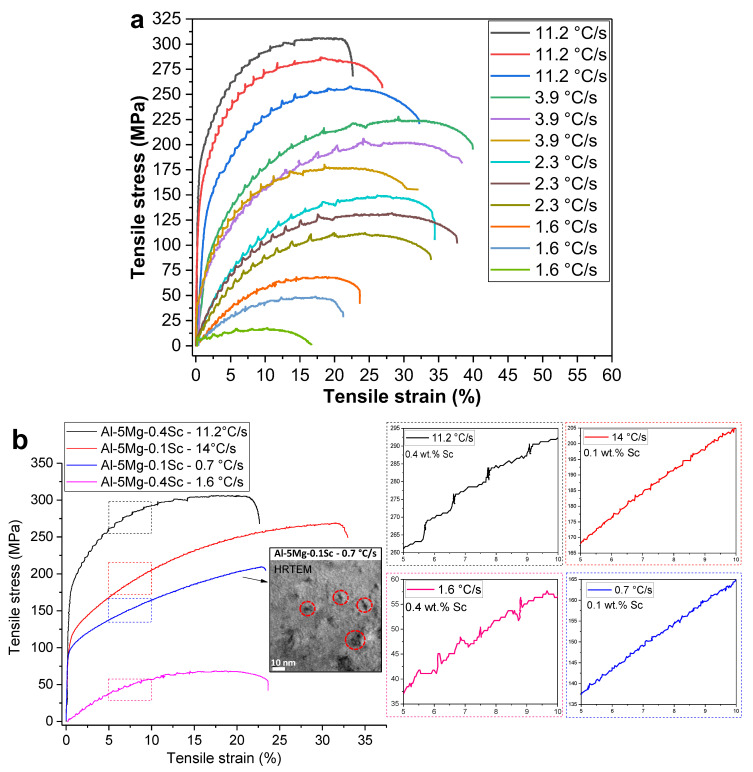
(**a**) Engineering stress–strain diagrams showing all curves for the Al-5Mg-0.4Sc alloy at different cooling-rate conditions, along with a (**b**) comparison between diagrams obtained for samples at high and low cooling rates for the Al-5Mg-0.1 And 0.4Sc alloys. The spherical or nearly spherical Al_3_Sc nanoprecipitates present in the Al-5Mg-0.1Sc alloy solidified at 0.7 °C/s are indicated by dashed red circles in the HRTEM image inserted in (**b**) [complementary data from the Al-5Mg-0.1Sc alloy from reference [14]].

Figure 16a depicts some typical stress–strain diagrams of the Al-5Mg-0.4Sc alloy at four distinct positions in the ingot, corresponding to different cooling rates. Analysis of the curves clearly shows that the highest cooling rate positions yielded superior tensile properties. The highest ultimate tensile strength (306 MPa) was achieved at one corresponding to the fastest cooling rate (11.2 °C/s), with an elongation of 22.6%. The lowest tensile strength was 260 MPa while the maximum elongation reached 32.2%. Considering the triplicate results for this cooling rate, the ultimate tensile strength averaged 284.3 ± 23.1 MPa, and the maximum elongation averaged 27.2 ± 4.8%, reflecting the variability inherent to this condition. With decreasing cooling rates, the mechanical behavior showed a clear transition consistent with the evolving microstructure in Figure 4. For the specimen solidified at 3.9 °C/s, the average ultimate tensile strength was 204.7 ± 24.0 MPa, with a maximum elongation of 36.8 ± 4.1%, indicating that reduced cooling promoted ductility at the expense of strength. At 2.3 °C/s, this trend continued, with the tensile strength decreasing to 131.3 ± 19.0 MPa, while the maximum elongation remained relatively high (35.3 ± 2.1%). In the slowest-cooled condition (1.6 °C/s), tensile strength dropped sharply to 45.0 ± 25.4 MPa, although the elongation still reached 23.2 ± 6.3%, as can be seen in Figure 16a. The TEM results revealed that, under the lowest solidification rate condition, larger and incoherent precipitates were formed relative to the α-Al matrix, together with locally continuous Sc-rich networks. These microstructural features promote stress concentration and interfacial decohesion at the Al_3_Sc/Al-rich matrix boundaries, likely enhancing the susceptibility to premature fracture under relatively low stresses. This interpretation is consistent with the pronounced loss in strength observed for this cooling-rate condition.

Besides the contribution from nanoprecipitation, the mechanical behavior of the alloy is also affected by the solidification morphology. At higher cooling rates (15.3 °C/s), both dendritic and eutectic structures are finer, whereas slower solidification-stage cooling allows longer solute diffusion, leading to the coarsening of dendrite arms as well as the interdendritic eutectic (see Figure 4 and Figure 6). Since solute redistribution is more efficient under such conditions, larger eutectic zones with fewer interfaces are formed. This reduction in interface density decreases the obstacles to dislocation motion, explaining the lower tensile strength of samples solidified under reduced cooling rates.

Figure 16b demonstrates more precisely the Portevin-Le Chatelier (PLC) effects in the tensile curves across typical cooling rates. In this case, results from a more dilute Sc-containing alloy, Al-5Mg-0.1Sc, have been put together in Figure 16b for comparison purposes. It is important to note that the Al–5Mg–0.1Sc alloy contains the same Mg content but four times less Sc, and its Al_3_Sc nanoprecipitates tend to exhibit a more spherical morphology [2], as seen in the TEM image in Figure 16b, markedly different from that observed in the alloy with higher Sc content.

For the 11.2 °C/s sample, the Al–5Mg–0.4Sc alloy exhibited frequent and sharp serrations in the plastic domain of the curves, characteristic of pronounced dislocation–solute interactions as described by the PLC effect. In contrast, the Al–5Mg–0.1Sc alloy solidified at 14 °C/s showed minimal serration activity, suggesting a reduced PLC effect likely due to lower Sc content and limited dislocation pinning. For the Al–5Mg–0.4Sc alloy processed at 1.6 °C/s, serrations were less frequent but still evident, denoting a moderate PLC regime. Finally, the Al–5Mg–0.1Sc alloy sample solidified at 0.7 °C/s displayed similar serrations as those observed in the same alloy for the sample solidified at higher cooling rate. Consequently, Figure 16b reveals minimal tensile curve variations comparing both Al-5Mg-0.1Sc alloy samples but significant differences if compared the Al-5Mg-0.4Sc alloy samples. While Mg is primarily responsible for dislocation pinning via Cottrell atmospheres [45], Sc can modify this behavior, once considering that both alloys have the same Mg content. It depends on the distribution of Sc-containing particles, their size, and the amount of Sc in solid solution.

In the case of the four samples tested under tension, the conditions differ significantly. In the alloy containing 0.1 wt.% Sc, fine spherical or nearly spherical Al_3_Sc nanoprecipitates, averaging below 10 nm in diameter, were detected, as evidenced by the HRTEM image inset in Figure 16b [2]. In contrast, the samples from the 0.4 wt.% Sc alloy exhibited distinct behaviors depending on the solidification rate. Rapidly solidified samples (11.2 °C/s) showed a predominance of Sc in solid solution, while the slowly solidified samples (1.6 °C/s) displayed rod-like or branched nanometric Al_3_Sc precipitates that were fully incoherent with the α-Al matrix.

The presence of semi-coherent or coherent Al_3_Sc precipitates forming the Al–5Mg–0.1Sc alloy may inhibit dislocation glide, thereby suppressing or altering the characteristics of the PLC effect, such as reducing strain localization or changing the serration type. In the case of the Al–5Mg–0.1Sc alloy, a smoother stress–strain response can be seen in Figure 16b thanks to the smaller size and higher coherence of the precipitates. Moreover, in this case, Mg solid solution strengthening appears to be the primary resistance mechanism. However, in the Al-5Mg-0.4Sc alloy sample corresponding to a solidification rate of 1.6 °C/s, where precipitates are coarser and heterogeneously distributed dislocation glide paths can be localized, enhancing the PLC effect by promoting stress concentration. The higher-Sc alloy combines solid solution strengthening mechanisms with precipitation hardening via Al_3_Sc formation, more evidenced at slower cooling rates. Although solid solution strengthening in the sample solidified as 11.2 °C/s resulted in improved mechanical properties, the PLC effect is still observed due to the Mg in solution, together with Sc, interacting with the dislocations during loading.

The mechanism is indeed synergistic: the higher Sc content, when coupled with lower cooling rates, promotes substantial precipitate coarsening, which enhances internal stress heterogeneity and thereby intensifies the PLC effect. This interpretation is reinforced by the fact that the Mg content is the same in both alloys, indicating that the observed behavior arises predominantly from the Sc-dependent precipitation evolution rather than from differences in Mg in solution.

The strengthening effect of Al_3_Sc nanoprecipitates was evaluated for two representative compositions. In the Al–5Mg–0.1Sc alloy, precipitates with an average size of ~5 nm and a volume fraction of ~0.2% are expected to be coherent and ordered. Under these conditions, strengthening is governed by shearing mechanisms associated with modulus hardening; coherency strengthening; and order strengthening [43]. Using a shear modulus, G, of 26 GPa, a Burgers vector, b, of 0.286 nm, a Taylor factor, M, of 3.06, a Poisson coefficient of 0.345, an APB (antiphase boundary) energy of ~0.15 J/m^2^, a modulus mismatch between Al and the Al_3_Sc, ΔG, of 68GPa, and a lattice misfit strain of ~0.92 according to TEM measurements, the estimated strengthening increment is on the order of 260 MPa.

In contrast, the Al–5Mg–0.4Sc alloy contains precipitates with an average size of ~40 nm and a volume fraction of ~1%. At this scale, the particles are no longer predominately shearable and are bypassed by dislocations through the Orowan looping mechanism. Using the Orowan stress formulation, the strengthening increment is reduced to approximately 50 MPa.

The lattice misfit between the Al_3_Sc precipitates and the α-Al matrix was determined from the lattice parameters shown in Figure 11. The Orowan stress increment was then calculated according to Equation (2), considering the average particle size and interparticle spacing measured experimentally.

(2) $${∆G}_{or}=M\frac{0.4Gb}{\pi \lambda }\frac{ln(\frac{2\bar{r}}{b})}{\sqrt{1-\upsilon }}$$

These distinct strengthening levels are consistent with the experimental tensile results, where the ultimate tensile strength of the Al–5Mg–0.1Sc alloy (268 MPa) exceeds that of the Al–5Mg–0.4Sc alloy (68 MPa), as shown in Figure 16b.

**Figure 17:**
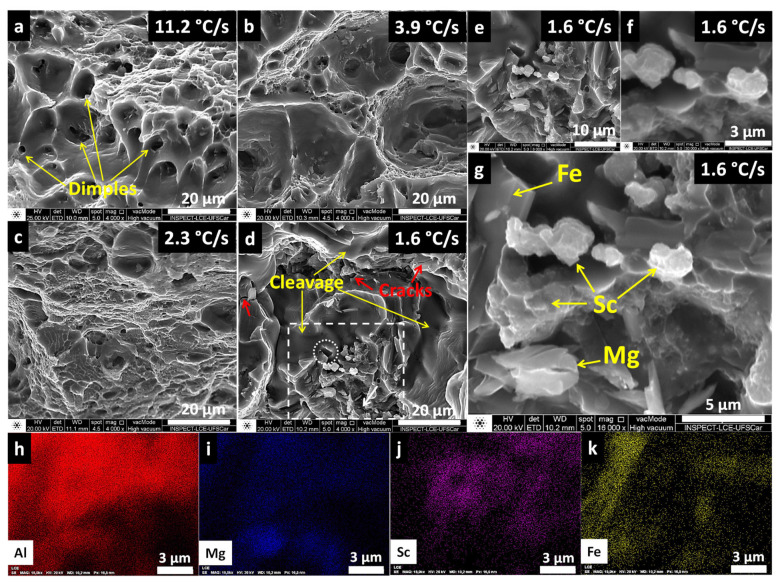
Fracture surface analysis of Al-5Mg-04Sc alloy tested under different cooling rates. (**a**–**d**) SEM/SE fractographs showing ductile-to-brittle transition with decreasing cooling rate (11.2 to 1.6 °C/s); (**e**–**g**) High-magnification views of brittle fracture (1.6 °C/s); (**h**–**k**) EDS maps of (**g**) revealing Sc clusters at fracture boundaries.

To further elucidate the tensile strength behavior, fracture surfaces of the tested samples were analyzed using SEM/SE imaging and SEM/EDS mapping. Figure 17a–g present the fractographs. The highest cooling rate (11.2 °C/s, Figure 17a) exhibits a clear dimple-like pattern, a well-known characteristic of the ductile fracture mode. Conversely, the lowest cooling rate (1.6 °C/s, Figure 17d) shows smooth cleavage surfaces indicative of a brittle fracture—consistent with the tensile results. This is further evidenced by the presence of cracks (red arrows) and the poor adhesion of deleterious phases (Al_6_Fe) with the matrix, the latter of which is indicated by a dashed white circle in Figure 17d. Intermediate cooling rates (3.9 °C/s and 2.3 °C/s, Figure 17b,c) display mixed fracture modes, with dimple density decreasing as the cooling rate decreases. In terms of mechanical properties, both exhibited similar tensile strain values compared with each other; however, with the decrease in the cooling rate, the tensile strength decreased. This can be associated with a greater predominance of cleavage and a lower density of dimples.

Figure 17e–g provide higher-magnification views of a region outlined by a white dashed rectangle in Figure 17d. This region is surrounded by a cleavage area. Sc-rich particles were observed in this central outlined area, in addition to Fe-rich particles (likely Al_6_Fe) and others rich in Mg (potentially Al_3_Mg_2_), as indicated in Figure 17g. EDS mapping (Figure 17h–k) of the fracture region in Figure 17g revealed Sc clusters (Figure 17j). These clusters, which can be observed in detail in Figure 17f (30,000×), are larger in size and likely formed due to the combined effects of high Sc content, low cooling rates, and grain boundary energy accumulation, originating from primary precipitation during solidification.

## 4. Conclusions

Directional solidification revealed steep thermal gradients and a strong inverse correlation between cooling rate and position along the ingot, enabling the extraction of samples with controlled microstructural length scales. Even under unidirectional heat flow, 0.4 wt.% Sc generated fully equiaxed α-Al grains through strong grain refinement, while 0.1 wt.% Sc produced predominantly columnar grains under similar conditions. TEM/STEM analyses indicated that high cooling rates (~15 °C/s) hinder Al_3_Sc formation because Sc diffusion is limited, the available time is short, and Mg may impose solute drag, whereas slow cooling (~1.3 °C/s) induces the development of coarser rod-like or branched precipitates and continuous Sc-rich networks, a response linked to alternative diffusion pathways during solid-state cooling. Faster cooling (~11 °C/s) also yielded higher tensile strength (up to 306 MPa) due to greater Sc retention in solid solution, finer overall microstructure, and reduced precipitate coarsening. The PLC effect becomes more evident in the high-Sc alloy across both cooling extremes, driven by Mg–dislocation interactions and influenced by Sc content, precipitate size, morphology, and spatial distribution. Moreover, the Al–5Mg–0.1Sc alloy contains small, coherent, highly regular Al_3_Sc precipitates (<10 nm) that minimize serrated flow, whereas the 0.4 wt.% Sc alloy develops rod-shaped or branched precipitates under slow cooling, enhancing strain localization.

## Figures and Tables

**Table 1:** EDS point analysis corresponding to the EDS points in Figure 7 and Figure 8.

Element	Matrix	1	2	3	4	5	6	7	8
	at.%								
**Al**	**94.1**	**84.1**	**95.7**	**92.5**	**90.0**	**86.8**	**82.6**	**67.6**	**84.2**
**Mg**	**5.6**	**13.3**	**4.0**	**7.3**	**8.7**	**13.1**	**16.4**	**13.5**	**12.6**
**Sc**	**0.3**	**0.4**	**0.3**	**0.2**	**0.1**	**0.0**	**0.1**	**0.0**	**0.5**
**Fe**	**0.0**	**2.2**	**0.0**	**0.0**	**0.0**	**0.0**	**0.3**	**14.6**	**0.8**
**Si**	**-**	**-**	**-**	**-**	**1.2**	**0.1**	**0.6**	**4.3**	**0.4**

## Data Availability

Data presented in this study are available on request from the corresponding author.
